# PAC1 receptor agonist maxadilan alleviates corneal inflammation in a murine model of LPS-induced ocular inflammation

**DOI:** 10.1371/journal.pone.0353866

**Published:** 2026-07-20

**Authors:** Andras Dobos, Balazs Meresz, Inez Bosnyak, Dorottya Molitor, Bela Kocsis, Edina Szabo, Dora Reglodi, Ildiko Vajda, Tamas Atlasz, Alexandra Vaczy

**Affiliations:** 1 Department of Anatomy, HUN-REN-PTE PACAP Research Team, Centre for Neuroscience, University of Pecs Medical School, Pecs, Hungary; 2 Kiseri Veterinary and Laser Ophthalmology Clinic, Szentes, Hungary; 3 Department of Medical Microbiology and Immunology, University of Pecs, Medical School, Pecs, Hungary; 4 Institute of Physical Education and Sports Science, University of Nyiregyhaza, Nyíregyhaza, Hungary; 5 Department of Sports Biology and Kinesiology; University of Pecs, Pecs, Hungary; Harvard Medical School, UNITED STATES OF AMERICA

## Abstract

Ocular inflammation is a leading cause of visual impairment and blindness worldwide. Systemic bacterial infections can spread hematogenously, resulting in endogenous endophthalmitis. These infections present significant diagnostic and therapeutic challenges due to their systemic origin and involvement of multiple ocular structures such as the retina and cornea. Pituitary adenylate cyclase-activating polypeptide (PACAP), acting through its selective receptor PAC1, exerts potent anti-inflammatory, anti-edema, and regenerative effects in various ocular conditions. This study aimed to assess the protective role of maxadilan, a selective PAC1 receptor agonist, in reducing corneal inflammation in a mouse model of lipopolysaccharide (LPS) -induced ocular inflammation. Mice received systemic LPS injections followed by intravitreal administration of maxadilan. Animals were monitored in vivo using optical coherence tomography (OCT) and tonometry. Post-mortem analyses included evaluation of cytokine expression profiles and routine corneal histological assessment. OCT revealed significantly reduced corneal edema and improved tissue structure with maxadilan administration. Additionally, maxadilan lowered intraocular pressure and reduced inflammatory cell infiltration into the aqueous humor. Cytokine profiling showed decreased expression of critical pro-inflammatory mediators such as GM-CSF, IL-6, MIP-1a, TNF-a, and TREM-1 in the maxadilan-treated group. Histological analyses confirmed that maxadilan preserved normal corneal architecture and effectively prevented pathological keratinization compared to untreated controls. These findings suggest that PAC1 receptor activation has therapeutic potential for controlling inflammation and maintaining corneal health in ocular inflammation.

## Introduction

The cornea is essential for protecting the eye and maintaining proper vision [1]. Injuries or pathological conditions in the eye, including untreated chronic inflammation, can lead to blindness and visual impairment. Endogenous endophthalmitis mainly affects the posterior segment of the eye, but when the condition is severe or sustained, the anterior segment can also become involved, potentially leading to chronic corneal edema, stromal fibrosis, neovascularization, and keratinization, which collectively increase the risk of permanent corneal opacity and vision impairment [1,2].

Ocular inflammation promotes the infiltration of immune cells into the eye’s tissues, and when this response becomes prolonged or remains untreated, it can lead to irreversible corneal damage [3,4]. In particular, the migration of inflammatory cells into the anterior chamber can disrupt corneal structure, triggering stromal remodeling that may result in thinning of the cornea and, in advanced cases, perforation [5]. The impairment of corneal transparency and permanent vision loss requires immediate medical attention and appropriate treatment to prevent complications [6]. While inhibiting cytokine expression during inflammation is an emerging therapeutic approach, antibiotics or steroids remain the primary treatment, where the potential adverse effects on the cornea should be weighed against their benefits [2,7].

Pituitary adenylate cyclase-activating polypeptide (PACAP) is a neuropeptide that belongs to the vasoactive intestinal polypeptide (VIP)/glucagon/secretin family of peptides [8–10]. PACAP is widely expressed throughout the body, with high levels in the central nervous system and ocular tissues. PACAP has been implicated in a variety of physiological processes [11]. PACAP interacts with three G protein-coupled receptor subtypes, which are known as PAC1 receptor (PAC1R), vasoactive intestinal polypeptide receptors 1 and 2 (VPAC1R, and VPAC2R). PAC1R has the highest affinity (∼1000-fold) for PACAP and functions as a PACAP-selective receptor, while VPAC1R and VPAC2R exhibit comparable affinities for PACAP and VIP peptides [12–14]. PACAP is known to have anti-inflammatory properties in several organs, including the eye [15–17], and has been implicated in the regulation of tear secretion and homeostasis, contributing to the maintenance of ocular surface health [18,19]. It has been shown to modulate anti-inflammatory responses, protect tissues against oxidative stress and promote tissue repair in various ocular conditions such as corneal wound healing [20–23]. PACAP is crucial for maintaining the physiological state of the corneal endothelium by enhancing cell viability, which preserves the integrity of the tight junctions in the cornea [24]. Intravitreal injection of exogenous PACAP has shown neuroprotective effects in a range of pathological conditions, such as glutamate-induced excitotoxicity, diabetic retinopathy, and carotid ligation-induced ischemic retinopathy [25–31]. Moreover, recent studies have demonstrated that PACAP, when administered in the form of eye drops, can rapidly penetrate through the ocular tissues and protect the retina in different diseases such as glaucoma or ischemia [32–37]. Recent research indicates that the significant protective effects of PACAP in different ophthalmological pathologies are PAC1R-mediated [38]. Therefore, a thorough investigation of this receptor is a priority for drug development. Maxadilan, a potent vasodilator peptide, was initially isolated from the salivary glands of the sand fly *Lutzomyia longipalpis.* The vasodilatory effect has been attributed to activation of the PAC1R, therefore, it is highly suitable for use as a selective PAC1R agonist in mammalian models to investigate the functional roles of this receptor [39–41].

Through the use of maxadilan, this research investigates the PAC1R-mediated protection against corneal inflammation triggered by lipopolysaccharide (LPS) -induced ocular inflammation in a murine model.

## Materials and methods

### Animals

Three-month-old adult male mice from the CD1 wild-type strain were used in the experiments (*n* = 102). These mice were housed in a controlled environment with regulated temperature and humidity following a 12-hour light/dark cycle. The animals were provided with unrestricted access to food and water throughout the duration of the study. All experimental procedures were conducted in accordance with institutional ethical guidelines and were approved under the following permission number: BA02/2000–01/2022.

### Endophthalmitis model and maxadilan treatment

The experimental timeline is summarized as follows: LPS administration and intravitreal treatment were performed on day 0, followed by acute assessments at 24 hours and chronic evaluations at 5 weeks. Mice were anesthetized using intraperitoneal (i.p.) injections of ketamine (90 mg/kg; Calypsol, Richter Gedeon, Budapest, Hungary) and xylazine (10 mg/kg; Sedaxylan, Dechra, Amsterdam, The Netherlands). *Escherichia coli* O55 LPS (6.0 mg/kg body weight) suspended in PBS was given (*n* = 56) through a single-dose intraperitoneal injection. As control, PBS was given to another group of mice (*n* = 46) as a single intraperitoneal injection. Before intravitreal treatment, Braunol solution (B. Braun Medical AG, Sempach, Switzerland) was applied to the corneal surface to prevent ocular infection. Half of the mice (*n = 28/* LPS group, *n = 23/* control group) were injected into the vitreous with a single dose of maxadilan solution (2 µL, 1 µM/µL), using a Hamilton syringe (33G), immediately after LPS administration. The remaining animals (n = *28/* LPS group, *n = 23/* control group) received the same volume of PBS. The following groups were formed based on the above description: Control + PBS (*n = 23*), Control + maxadilan (*n = 23*), LPS + PBS (*n = 28*), LPS + maxadilan (*n = 28*). To evaluate early inflammatory responses, morphological and physiological assessments were performed 24 hours after LPS administration. In this acute phase, in vivo spectral-domain OCT (SD-OCT) was used to monitor corneal structural changes, and intraocular pressure was measured at the same time point. Additionally, a subset of mice was euthanized at 24 hours, and corneal tissues were collected for cytokine array analysis to characterize early immune activation.

To assess longer-term morphological alterations associated with chronic inflammation, additional examinations were conducted 5 weeks after LPS injection. At this time point, corneal surface changes were evaluated using stereomicroscopy, and routine histology was performed to characterize stromal and epithelial remodeling processes.

### Optical coherence tomography (OCT)

For SD-OCT mice (*n* = 11/ in each control group, *n* = 21/ in each LPS group) were anesthetized with a single intraperitoneal injection of 2.5 μL/g of a mouse cocktail containing 5.5 mL physiological saline (B.Braun, Budapest, Hungary), 1 mL xylazine (20 mg/mL Sedaxylan, Dechra, Northwich, United Kingdom) and 3.5 mL ketamine (20 mg/ml Calypso, Richter Gedeon, Budapest, Hungary). Anesthesia was administered one day before the intervention (baseline measurement) and again 24 hours after LPS injection, when the inflammatory response reaches its peak. To ensure corneal lubrication during imaging, Systane Ultra lubricant eye drops (Systane, Alcon Hungaria Ltd., Budapest, Hungary) were applied to each eye every three minutes. OCT images were captured using the Bioptigen Envisu R-class Image Guided SD-OCT system (Leica Microsystem, Morrisville, NC, USA). The rectangular scans were conducted with specific parameters: 2000 A-scans/ 150 B-scans × 3 Frames/ each B-scan. A 10 mm anterior imaging lens was used to take these scans, covering a physical area of 1.8 mm × 1.8 mm on the tissue. For consistent and accurate OCT measurements in mice, the eyes were aligned such that the irises were positioned symmetrically across the optical axis, ensuring that the scan was centered on the central corneal region. To determine the thickness of central corneal layers in multiple cross-sectional images and to visualize a 3D model from the corneal structure, the Bioptigen software InVivoVue Diver (Leica Microsystem, Morrisville, NC, USA) was employed. For statistical analysis of the SD-OCT studies, the OriginPro 2018 (OriginLab Corporation, Northampton, MA, USA) statistical package was utilized.

### IOP measurement

Mice were handled and acclimatized for approximately 2 weeks (*n* = 5/ in each control group, *n* = 8/ in each LPS group). The baseline of IOPs was measured in both eyes using a rebound tonometer (Tonolab, Icare; Vantaa, Finland) the day before LPS injection. IOP was remeasured in conscious animals 24 hours after LPS injection. In order to prevent issues with circadian variation in IOP, measurements were taken in the afternoon at the same time of the day, starting between 1:30 p.m. and 2:30 p.m. For each eye, the mean value of three consecutive measurements was recorded. After measuring the IOP, the ocular surface was lubricated with artificial tears (Alcon Hungaria Ltd., Budapest, Hungary).

### Cytokine array analysis

Cytokine array analysis was conducted one day after the administration of LPS, using corneas from four mice per experimental group (*n = 16* per independent experiment). The experiment was repeated three times independently. Corneas were dissected and stored at −80°C until testing. The Proteome Profiler Mouse Cytokine Array Kit, Panel A from R&D Systems (Biomedica, Budapest, Hungary), was utilized for the analysis. The array operates on the principle of antibodies binding to the nitrocellulose membranes, following the manufacturer’s protocol. Samples were pooled and homogenized in PBS containing protease inhibitors. Triton X-100 was added after homogenization. Nitrocellulose membranes were subsequently blocked and incubated with a reconstituted detection antibody cocktail. Overnight incubation with 400 µg protein-containing homogenates was performed. Following the washing steps, streptavidin-horseradish peroxidase was added to the membranes. Membranes were exposed to a chemiluminescent detection reagent (Biosciences, Budapest, Hungary). Developed films were scanned, and the mean intensities of the dot blots representing different cytokines were calculated using ImageJ software (NIH, Bethesda, ML, USA).

### Keratitis score evaluation

An investigator, unaware of the treatments, evaluated the severity of the developed keratitis (*n* = 6 in each control group, *n* = 6 in each LPS group) using a stereomicroscope on the 5th week following LPS injection. The eyes were examined and photographed with a stereomicroscope (Leica EZ4, Leica Microsystem, Wetzlar, Germany). As previously described (Shioda et al., 2018), keratitis was scored as follows: 0 = no abnormalities observed; the cornea was clear without any signs of inflammation. 1 = mild corneal haze was present, with visible iris, 2 = moderate corneal flare, accompanied by moderate corneal haze and superficial punctate keratitis, 3 = severe damage and loss of corneal tissue were evident.

### Corneal histology

Mice were euthanized 5 weeks after LPS administration, and the eyes were enucleated (*n =* 2 in each group). Cornea samples were isolated from the eyeball and fixed overnight in 4% paraformaldehyde. After embedding in paraffin, the eyes were cut into 12 μm sections and stained with hematoxylin and eosin. Samples were mounted with DPX (Sigma, Merck, Budapest, Hungary) and covered with coverslips. Digital photographs of the central corneal region were taken with a Nikon Eclipse 80i fluorescence microscope (Nikon, Melville, NY, USA) with a 40x objective.

### Statistical analysis

Statistical analyses were performed using OriginPro 2018 (OriginLab Corporation, Northampton, MA, USA). Normality was assessed with the Kolmogorov–Smirnov test, followed by one-way ANOVA with either Fisher’s LSD or Holm–Šidák post hoc analysis, as appropriate. Pairwise comparisons were evaluated using two-sample t-tests. Data are presented as means ± SEM, with differences considered significant at p < 0.05.

## Results

### Optical coherence tomography (OCT)

In this study, we utilized maxadilan treatment to explore the potential anti-inflammatory function of the PAC1R in the cornea. We primarily analyzed structural alterations in the cornea, emphasizing central corneal thickness, during the acute phase of ocular inflammation. Furthermore, we also aimed to investigate signs of anterior chamber inflammation, specifically the notable cell infiltration into the aqueous humor. In the control group injected with PBS, both OCT B-scans and OCT 3D images demonstrated an intact corneal structure with no changes in the thickness or morphology, and the aqueous humor in the anterior chamber was also clearly apparent, as in the baseline measurements (Figs 1A and 2A and S1). The control + PBS group had similar corneal integrity as the maxadilan treatment alone (Figs 1B and 2B). After 24 hours of LPS injection, the corneas exhibited marked structural disturbances with significant thickening and irregularities in the tissues (Figs 1C, 1D and 2C, 2D). In the LPS + PBS-treated group, corneal edema was apparent, leading to the distortion of corneal layers. Moreover, the anterior chamber displayed large, irregular hyper-reflective cellular materials, suggestive of hypopyon (Figs 1C and 2C). The administration of maxadilan ameliorated the corneal structural changes caused by LPS (Figs 1D and 2D). The increase in corneal thickness was less detectable than in the LPS + PBS group, suggesting a decrease in corneal edema. Moreover, a noticeable reduction in cell infiltration was observed in the anterior chamber and the aqueous humor was more transparent compared to the LPS + PBS injected group, indicating a reduction in inflammatory activity (Figs 1C, 2D and 2C, 2D).

**Fig 1:**
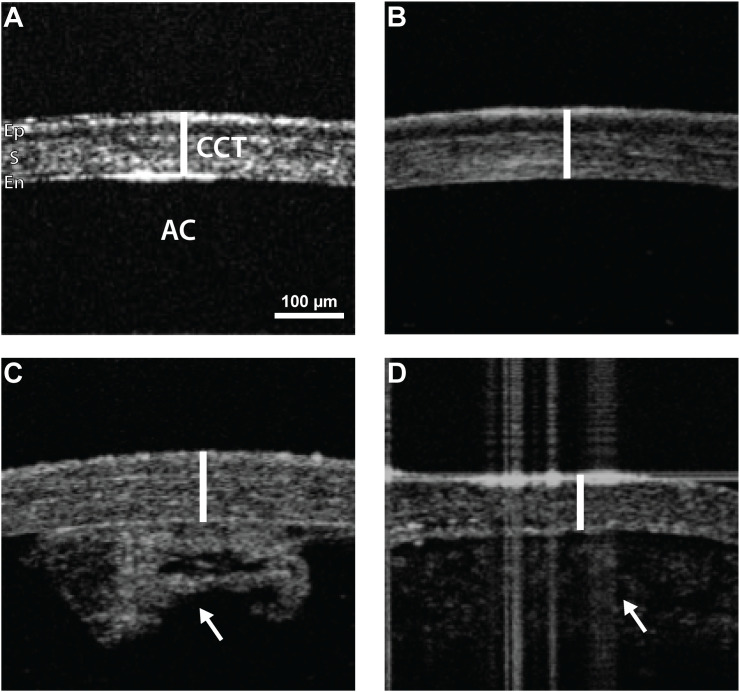
Demonstrative anterior segment OCT B-scan images reveal the structure of the central corneal area with part of the anterior chamber, recorded 24 hours after LPS administration. **A:** Control cornea following i.v.i. PBS. **B:** Control cornea, treated with i.v.i. maxadilan. **C:** LPS-injected mouse cornea treated with i.v.i. PBS or **D:** treated with i.v.i. maxadilan. The anterior chamber of the eye showed visible inflammation induced by LPS, as evidenced by a large, irregular hyper-reflective cellular material floating (white arrows), and this was markedly reduced in the LPS + maxadilan group. Moreover, the cornea showed more pronounced edema (vertical white bar indicates the measurement location) in the LPS + PBS group compared to the LPS group treated with maxadilan. These OCT images illustrate qualitative structural changes (white arrows); quantitative analyses of corneal thickness (vertical white bar) and inflammation are presented in later figures. Abbreviations: LPS: lipopolysaccharide, PBS: phosphate-buffered saline, i.v.i: intravitreal injection, AC: anterior chamber, CCT: central corneal thickness, Ep: epithelium, S: stroma, En: endothelium.

**Fig 2:**
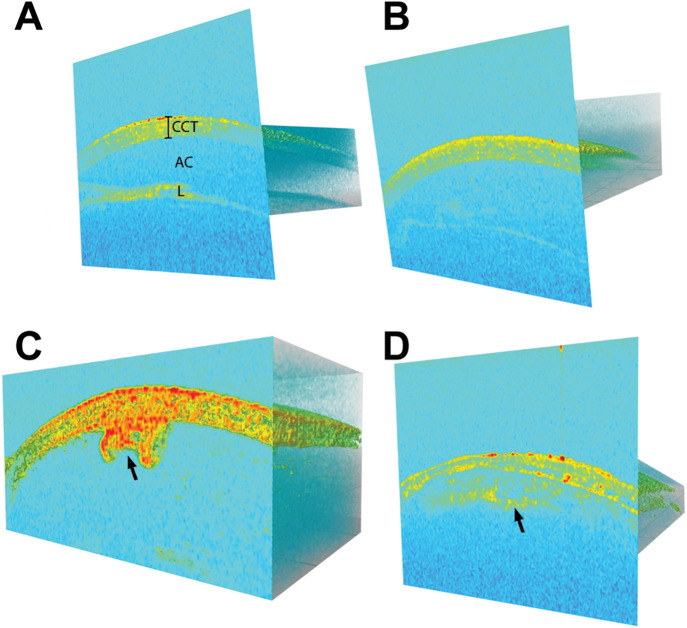
Representative image showing the 3D structure of the cornea based on B-scan images in different groups in mice, recorded 24 hours after LPS administration. These images illustrate the overall corneal architecture and anterior chamber changes 24 hours after treatment. Panels correspond to the following conditions: **A:** Control cornea, treated with i.v.i. PBS. **B:** Control cornea, treated with i.v.i. maxadilan. **C:** LPS-injected cornea treated with i.v.i. PBS or **D:** injected with i.v.i. maxadilan. LPS resulted in massive cellular material floating in the anterior chamber (black arrows) and induced corneal edema, which was more prominent in the LPS + PBS-treated group compared to the LPS + maxadilan ones. Abbreviations: LPS: lipopolysaccharide, PBS: phosphate-buffered saline, i.v.i: intravitreal injection, AC: anterior chamber, CCT: central corneal thickness, L: lens.

The observations from the B-scan images confirmed the statistical analysis of the central corneal thickness (Fig 3). The endotoxin effect led to a significant increase in total central corneal thickness in both groups after 24 hours. However, in the LPS + maxadilan-treated group, the central corneal thickness changes were significantly smaller compared to the LPS + PBS-received group (Fig 3).

**Fig 3:**
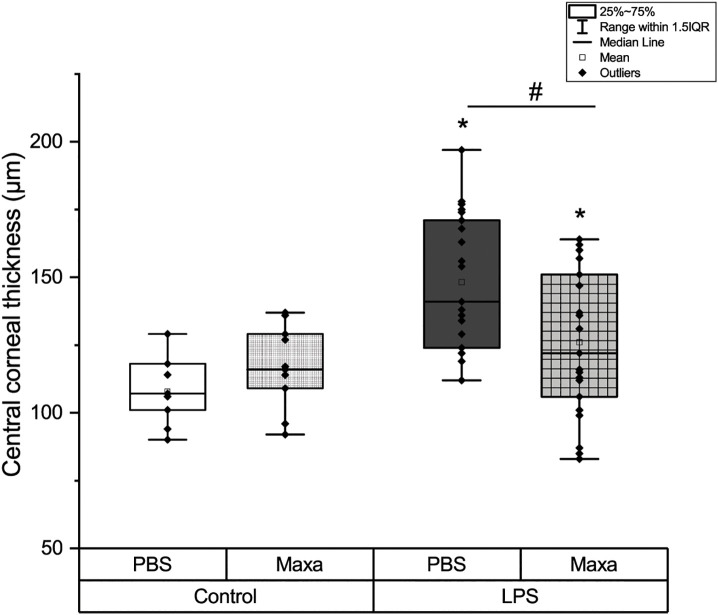
Quantitative comparison of the central corneal thickness in the investigated groups: Control + PBS, Control + Maxa, LPS + PBS, LPS + Maxa, measured 24 hours after LPS administration. LPS administration significantly increased the central corneal thickness (CCT) compared to controls (Control + PBS: 107.72 ± 3.43 µm vs. LPS + PBS: 148.28 ± 5.4 µm; p < 0.01), corresponding to a 37.64% increase in thickness. Maxadilan treatment significantly reduced this LPS-induced edema (LPS + Maxa: 126.09 ± 5.98 µm; p < 0.01 vs. LPS + PBS), resulting in a 22.19 µm (14.96%) decrease in corneal thickness. No significant difference was observed between the two control groups (Control + PBS vs. Control + Maxa; p = 0.37). On the graph, the top of the box denotes the 75^th^ percentile, the bottom of the box signifies the 25^th^ percentile, and the central line represents the 50^th^ percentile value. The whiskers extend to the highest and lowest values that do not qualify as outliers or extremes. Statistical analysis based on two-way ANOVA with Fisher’s LSD. Data shown *p < 0.05 compared to the Control + PBS, #p < 0.05 between the two LPS groups. Sample sizes were Control + PBS (n = 11), Control + Maxa (n = 11), LPS + PBS (n = 21), and LPS + Maxa (n = 21). Abbreviations: Maxa: maxadilan, LPS: lipopolysaccharide, PBS: phosphate-buffered saline.

### Intraocular pressure (IOP) measurement

IOP was measured in the experiment in all four study groups. There were no variations in IOP levels between the two control groups. However, a notable difference in IOP was detected after LPS injection in both treated groups, showing a greater increase in the PBS-injected group compared to the maxadilan-treated ones (Fig 4).

**Fig 4:**
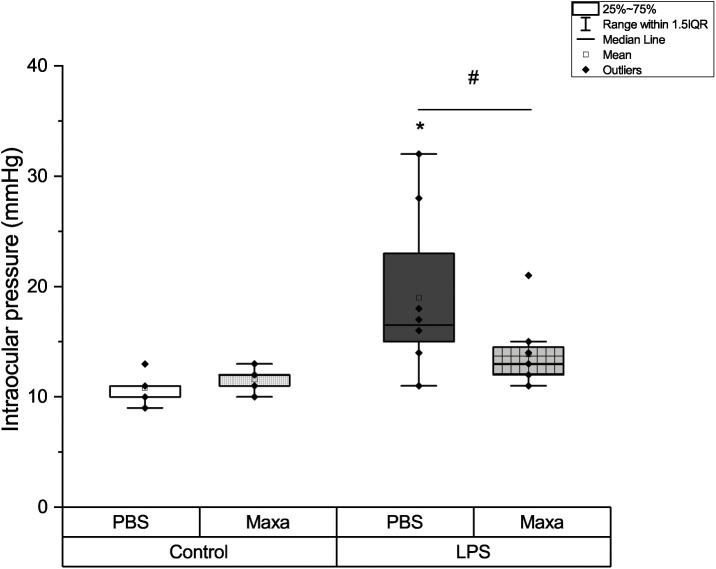
IOP values of the four study groups, measured 24 hours after LPS administration. Mean IOP values were 10.8 ± 0.66 mmHg in the Control + PBS group, 11.6 ± 1.14 mmHg in the Control + Maxa group, 19.0 ± 2.54 mmHg in the LPS + PBS group, and 13.88 ± 1.1 mmHg in the LPS + Maxa group. LPS + PBS increased IOP by 75.93% compared with Control + PBS, whereas maxadilan reduced the LPS-induced elevation by 26.97% (5.12 mmHg difference). Statistical analysis demonstrated that the increase in IOP in the LPS + PBS group compared with the Control + PBS group was significant (p = 0.03), and the reduction in IOP in the LPS + Maxa group compared with the LPS + PBS group was also significant (p = 0.04). *p < 0.05 compared to Control + PBS; #p < 0.05 between the two LPS groups. On the graph, the top of the box denotes the 75^th^ percentile, the bottom of the box signifies the 25^th^ percentile, and the central line represents the 50^th^ percentile value. The whiskers extend to the highest and lowest values that do not qualify as outliers. Sample sizes were Control + PBS (n = 5), Control + Maxa (n = 5), LPS + PBS (n = 8), and LPS + Maxa (n = 8). Abbreviations: PBS: phosphate-buffered saline, Maxa: maxadilan, LPS: lipopolysaccharide.

### Cytokine array analysis

Following LPS treatment (Figs 5A and 5B), the levels of several cytokines increased notably in both groups. Specifically, complement component C5a (C5/C5a), granulocyte colony-stimulating factor (G-CSF), granulocyte macrophage colony-stimulating factor (GM-CSF), soluble intercellular adhesion molecule-1 (s-ICAM1), interleukin 1 receptor antagonist (IL-1ra), interleukin 2 (IL-2), interleukin 3 (IL-3), interleukin 5 (IL-5), interleukin 6 (IL-6), interleukin 7 (IL-7), interleukin 13 (IL-13), interleukin 23 (IL-23), interferon gamma-induced protein 10 (IP-10), keratinocyte-derived chemokine (KC), macrophage colony-stimulating factor (M-CSF), monocyte chemoattractant protein-1 (JE), monocyte chemotactic protein 5 (MCP-5), macrophage inflammatory protein 1 alpha (MIP-1a), macrophage inflammatory protein 2 (MIP-2), regulated upon activation normal T cell expressed and presumably secreted (RANTES), tissue inhibitor of metalloproteinase 1 (TIMP-1), tumor necrosis factor alpha (TNF-a), and triggering receptor expressed on myeloid cells 1 (TREM-1) showed increased expression in LPS-induced corneal inflammation compared to control groups (Fig 5 A, 5B). In the maxadilan-treated LPS group, the expressions of GM-CSF, IL-6, MIP-1a, TNF-a, and TREM-1 were significantly lower compared to those in the LPS + PBS group (Fig 5 A-5G). However, there were no significant changes observed in the expression levels of B lymphocyte chemoattractant (BLC), chemokine C-C motif ligand 1 (I-309), C-C motif chemokine 11 (Eotaxin), interferon-gamma (IFN-γ), interleukin 1 alpha (IL-1a), interleukin 1 beta (IL-1b), interleukin 4 (IL-4), interleukin 10 (IL-10), interleukin 12 isoform 70 (IL-12p70), interleukin 16 (IL-16), interleukin 17 (IL-17), interleukin 27 (IL-27), interferon-inducible T-cell alpha chemoattractant (I-TAC), monokine induced by gamma (MIG), macrophage inflammatory protein 1 beta (MIP-1b), stromal cell-derived factor 1 (SDF-1), and thymus and activation-regulated chemokine (TARC) (Fig 5A).

**Fig 5:**
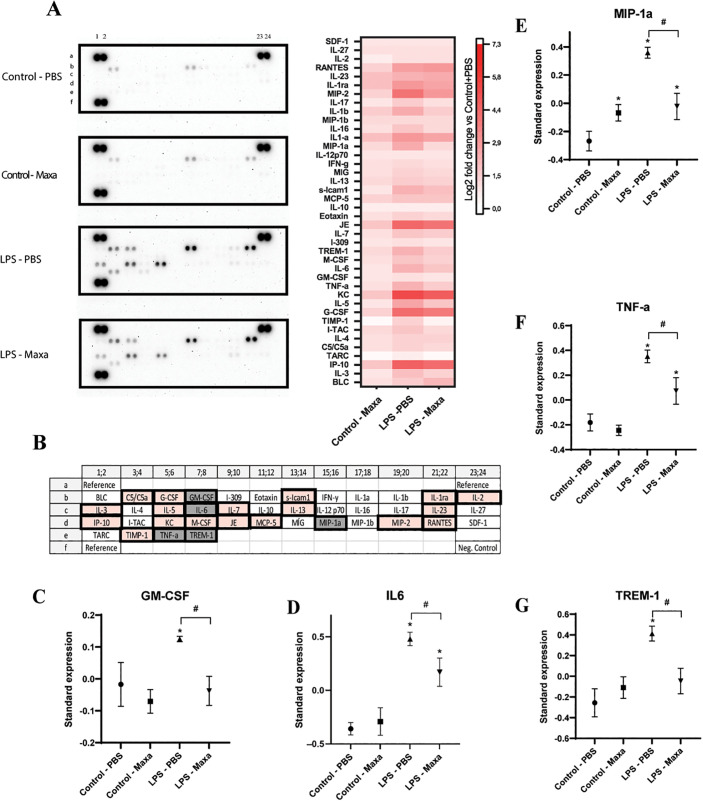
Effects of maxadilan treatment on cytokine production in control and LPS-treated corneas, measured 24 hours after LPS or PBS administration, representing the acute inflammatory phase. **A:** Representative panels display cytokine arrays from the initial run of Control + PBS, Control + Maxa, LPS + PBS, and LPS + Maxa corneas, together with a heatmap summarizing cytokine expression patterns across the experimental groups relative to the Control + PBS group. **B:** The table indicates the examined cytokines in each box, highlighting with background colors those that significantly changed after LPS. Additionally, the table marks cytokines with black rectangles and a gray background if their expression was significantly lower in the LPS maxadilan-treated group compared to the LPS PBS-treated group. Cytokines were measured with Protein Array Analyzer in ImageJ Version 1.54 software, and the results represent the average of three independent replicates. Graphs **C-G:** illustrate the standardized expression of five different cytokines /**C:** GM-CSF, **D:** IL-6, **E:** MIP-1a, **F:** TNF-a, **G:** TREM-1/ demonstrating the amelioration of LPS-induced inflammation by maxadilan in cornea samples. Data are expressed as mean ± SEM. Statistical analysis based on one-way ANOVA with Holm–Šidák post hoc. *p < 0.05 compared to the Control-PBS group, #p < 0.05 between the two LPS groups. Quantitatively, LPS markedly increased the expression of numerous analyzed cytokines, whereas maxadilan significantly reduced these elevations. Compared with the LPS + PBS group, maxadilan treatment reduced GM-CSF by 34.49% (p = 0.01), IL-6 by 55.81% (p = 0.01), TNF-a by 46.18% (p < 0.01), MIP-1α by 58.07% (p < 0.01), and TREM-1 by 144.64% (p < 0.01). These results demonstrate a consistent and statistically significant attenuation of LPS-induced corneal inflammation. Sample sizes were Control + PBS (n = 12), Control + Maxa (n = 12), LPS + PBS (n = 12), and LPS + Maxa (n = 12). Data were obtained from three independent biological replicates, each using pooled corneas from four mice per group. Abbreviations: PBS: phosphate-buffered saline, Maxa: maxadilan, LPS: lipopolysaccharide; GM-CSF: granulocyte macrophage colony-stimulating factor, IL-6: interleukin 6, MIP-1a: macrophage inflammatory protein-1 alpha, TNF-a: tumor necrosis factor alpha, TREM-1: triggering receptor expressed on myeloid cells 1.

### Keratitis score evaluation

Using a stereomicroscope, we monitored the impact of endotoxin-induced chronic inflammation on the surface of the mouse corneas up to the 5^th^ week following intraperitoneal LPS injection. In several cases, hypertrophy of the corneal epithelial cells was observed, which led to an opaque-like cloudiness in the eyes. These changes were associated with the chronic inflammation stage, ultimately resulting in varying degrees of corneal keratinization (Fig 6A). In more severe cases, angiogenesis was also observed on the corneal surface, and the central region of the cornea exhibited completely opaque thick plaques. To assess the degree of corneal keratinization, we categorized it into four stages using a stereomicroscope, ranging from Grade 0 (normal) to Grade 3 (hypertrophy of the surface, keratinization, and angiogenesis) (Fig 6A). At the end of the experiment, 100% of the mice in the control groups had intact corneas. After five weeks of inflammation, 33.3% of the mice in the LPS + maxadilan group maintained Grade 0 corneas, whereas only 16.6% of the mice in the LPS + PBS group achieved the same outcome (Fig 6B). In the LPS + maxadilan-treated group, 50% of animals exhibited the initial stage of keratinization (Grade 1), considerably more than the 16.6% observed in the LPS + PBS group (Fig 6B). The prevalence of individuals in the second stage of keratinization (Grade 2) was similar (about 17%) in both groups that received endotoxin injections (Fig 6B). Approximately 50% of the population showed the most severe keratinization grading (Grade 3) in the LPS + PBS-treated group (Fig 6B).

**Fig 6:**
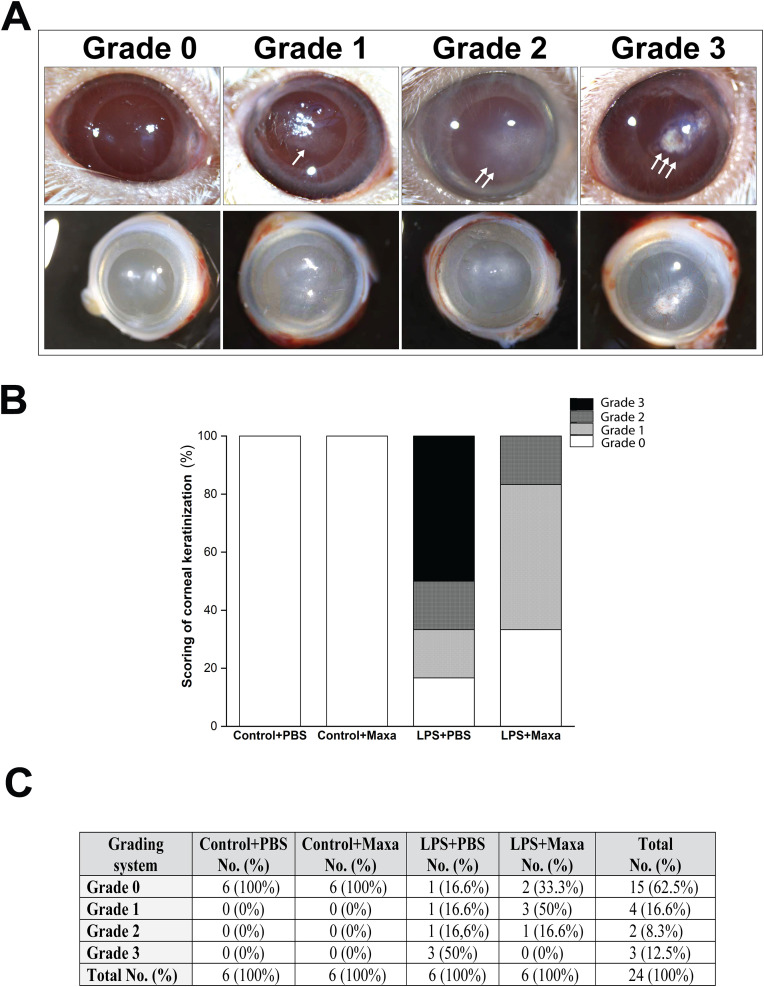
Scoring of corneal keratinization in all study groups, evaluated 5 weeks after LPS or PBS administration, representing the chronic phase of the experiment. **A:** The stereomicroscope image illustrates the four stages of keratinization: Grade 0 (normal), Grade 1 (mild), Grade 2 (moderate), and Grade 3 (severe). The number of white arrows indicates the severity of the developed keratitis in the picture. **B:** The graph displays the distribution of the animals belonging to different groups according to the keratinization scoring system in percentage distribution. In the LPS + PBS group, 50% of the eyes developed Grade 3 keratinization, whereas maxadilan treatment reduced this to 0% (complete prevention of severe keratinization). Furthermore, the proportion of Grade 0 corneas increased from 16.6% in the LPS + PBS group to 33.3% in the LPS + Maxa group. **C:** Group-wise distribution of animals by keratinization grade**.** Overall, maxadilan shifted the keratinization profile toward milder grades, reducing severe keratinization and increasing the proportion of normal corneas. Sample sizes were Control + PBS (n = 6), Control + Maxa (n = 6), LPS + PBS (n = 6), and LPS + Maxa (n = 6). Abbreviations: PBS: phosphate-buffered saline, Maxa: maxadilan, LPS: lipopolysaccharide, No. (%): animal number (percentage).

### Corneal histology

Hematoxylin and eosin-stained paraffin sections from both control groups (Fig 7A, 7B) showed healthy corneal structures 5 weeks after LPS injection. The corneal structure, including collagen remodeling, was altered by i.p. endotoxin injection, with the LPS + PBS-treated group experiencing a more severe effect than the LPS + maxadilan group (Fig 7C, 7D). Furthermore, the LPS + PBS-treated group had more irregularly arranged epithelial cells, while the maxadilan-treated individuals showed the same conditions as the control groups.

**Fig 7:**
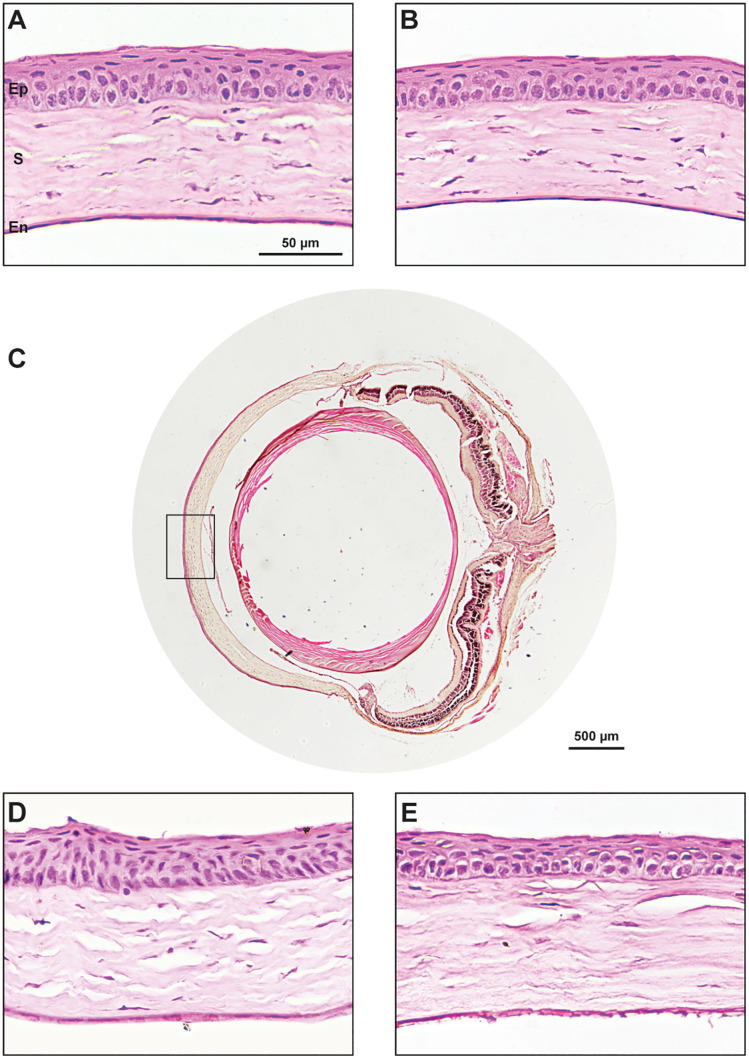
Representative photomicrographs illustrate hematoxylin and eosin-stained paraffin sections of the cornea 5 weeks after i.p. LPS injection in mice, representing the chronic phase of the experiment. **A:** Control group exhibited the normal structure of the cornea. **B:** Similar structure was observed to the controls after maxadilan injection. **D:** The LPS + PBS group exhibits dispersed collagen bundles in the stroma, and irregularly arranged cells in the epithelium were found, which resulted in irregular corneal surface. **E:** Treatment with maxadilan (LPS + Maxa) results in decreased collagen dispersion and improved preservation of the epithelial cell layer compared to the LPS + PBS group. **C:** Cross-section image of an eye captured using manual large image mode with 4x objective. The square indicates the central corneal region, which was subsequently imaged using 40x objective for detailed analysis. Sample sizes were Control + PBS (n = 2), Control + Maxa (n = 2), LPS + PBS (n = 2), and LPS + Maxa (n = 2). Abbreviations: i.p.: intraperitoneal, LPS: lipopolysaccharide, PBS: phosphate-buffered saline, Ep: epithelium, S: stroma, En: endothelium.

## Discussion

Using maxadilan as a selective PAC1R agonist, we present evidence that PAC1R activation contributes to corneal protection during inflammation triggered by LPS-induced ocular inflammation. All microbial infections significantly contribute to corneal inflammation, a primary cause of corneal opacification and blindness worldwide. Gram-negative bacterial infections are among the most common causes of systemic inflammation of the body, and this can also trigger severe ocular surface inflammation in the corneal tissue, thereby exacerbating and prolonging pathological processes of the eye. Moreover, LPS can activate the complement system and trigger the release of numerous inflammatory cytokines. In addition, the effective treatment of Gram-negative bacterial infections faces increasing challenges due to rising antibiotic resistance [42,43].

In our study, systemic administration of *Escherichia coli* LPS initiated ocular inflammation, which involved inflammation of the corneal tissue. During the initial phase of this inflammation, resembling clinical scenarios [44–46], we observed severe eye inflammation induced by LPS. This was characterized by heightened light sensitivity, increased tearing, eye discharge, and redness. Moreover, animals afflicted with chronic corneal inflammation without any treatment exhibited elevated eye pressure, mirroring the findings observed by Eliott and colleagues among human patients, which further indicates the endogenous intraocular inflammation and the presence of corneal edema [47,48]. The neuropeptide PACAP and its specific receptor PAC1 are distributed across various layers of the eye, including the cornea, where the PAC1R is present in both epithelial and endothelial layers. Our research demonstrated that the LPS administration increased IOP by 75.93% compared with controls, while maxadilan reduced this elevation by 5.12 mmHg (26.97% decrease), resulting in an IOP that was 1.37-fold lower than in untreated LPS animals. This finding is consistent with our earlier studies, which also documented the intraocular pressure-lowering effects of PACAP treatment in a glaucoma model [35]. The precise mechanism underlying PACAP’s pressure-lowering effects remains unclear, but it is speculated that PACAP can achieve this by regulating the production and the flow of the aqueous humor. This modulation likely involves the regulation of cAMP levels and the small GTPase Ras homolog family member A, mediated through the PAC1R [49–52]. The PAC1R helps to lower IOP not only by controlling the production of aqueous humor but also through additional mechanisms involving inflammation reduction and swelling prevention. It should be noted that corneal edema may influence rebound tonometry measurements, potentially leading to an overestimation of intraocular pressure. In addition, inflammatory cell infiltration into the anterior chamber may also contribute to altered IOP values by affecting aqueous humor dynamics. Therefore, the observed IOP changes in our study likely reflect a combination of true inflammatory effects and measurement-related factors. Consistent with this, LPS increased central corneal thickness by 37.64% (107.72 µm → 148.0 µm), while maxadilan reduced this LPS-induced swelling by 22.19 µm (14.96%), resulting in a cornea that was 1.17-fold thinner than in untreated LPS eyes. Activation of PAC1R signaling has been reported to reduce leukocyte infiltration in inflammatory conditions, which may contribute to the attenuation of corneal edema observed in our study [53]. PACAP’s edema-reducing effects have been described in several tissues and involve stabilization of barrier integrity and regulation of tissue fluid balance through AC–cAMP–PKA signaling and related pathways. These mechanisms may contribute to the reduced corneal edema observed in our model [54–59].

Several studies highlight the potential of PAC1R in ocular conditions linked to inflammatory dysregulation [60,61]. The PAC1R plays a crucial role in modulating inflammatory responses by reducing pro-inflammatory cytokine production, enhancing anti-inflammatory cytokine expression, and maintaining tissue integrity [41]. Activation of PAC1R by maxadilan triggers intracellular signaling pathways, primarily involving the cAMP/PKA and MAPK/CREB cascades, which are known to inhibit NF-κB activation and downstream cytokine production. Through these mechanisms, PAC1R activation can suppress excessive inflammatory signaling, reduce immune cell recruitment, and contribute to the preservation of corneal structure during inflammatory conditions. Endotoxins such as LPS powerfully activate the innate immune system primarily through toll-like receptor 4 signaling [62]. This cascade activates nuclear factor-kappa B, which in turn increases the transcription of various proinflammatory cytokines, including IFN-γ, TNF-a, IL-2, IL-4, IL-8, IL-6, IL-10, IL-1β, and VEGF-a [63,64]. In our study evaluating the impact of the PAC1R agonist maxadilan, we observed similar cytokine expression in the corneas following LPS injection. Compared to the untreated LPS group, maxadilan significantly reduced the levels of GM-CSF, MIP-1a, IL-6, TNF-a, and TREM-1, indicating its protective role in alleviating corneal inflammation. These cytokines are key components of interconnected inflammatory signaling networks, particularly those regulated by NF-κB and Toll-like receptor pathways. TNF-α and IL-6 act as central amplifiers of inflammatory cascades, while MIP-1α drives leukocyte recruitment and TREM-1 further enhances TLR-mediated responses, creating a positive feedback loop that sustains inflammation [65]. The simultaneous attenuation of these mediators by maxadilan suggests a coordinated suppression of these signaling networks rather than isolated cytokine effects. Mechanistically, PAC1R activation is known to engage intracellular pathways such as cAMP/PKA and MAPK/CREB signaling, which can inhibit NF-κB activation and downstream cytokine production [66]. Through this integrated pathway modulation, PAC1R activation likely contributes to reduced immune cell infiltration, decreased corneal edema, and preservation of tissue structure. Maxadilan significantly reduced the LPS-induced increase in multiple pro-inflammatory cytokines: GM-CSF decreased by 34.49% (1.39-fold), IL-6 by 55.81% (1.61-fold), TNF-α by 46.18% (1.73-fold), MIP-1α by 58.07% (1.98-fold), and TREM-1 by 144.64% (1.73-fold). GM-CSF is essential for the activation and differentiation of granulocytes and macrophages; during corneal inflammation, it promotes immune cell infiltration, which can lead to tissue damage and delayed healing. Previous studies have demonstrated that excessive GM-CSF expression exacerbates corneal inflammation and contributes to stromal degradation [67–69]. Thus, by lowering GM-CSF levels, maxadilan may support a controlled immune response that minimizes tissue damage while preserving antimicrobial activity. MIP-1a, a chemokine that recruits neutrophils and macrophages to inflamed tissues, has been implicated in severe inflammatory responses in corneal infection, contributing to corneal opacity and immune cell infiltration [70]. The reduction of MIP-1a expression by maxadilan indicates a diminished chemotactic response, which may help reduce immune-mediated tissue damage and maintain corneal transparency. Similarly, TREM-1 enhances inflammatory responses by boosting cytokine production and immune cell activation. Increased TREM-1 expression has been associated with exacerbated inflammatory responses in infectious conditions, including corneal inflammation, where it contributes to the amplification of Toll-like receptor–mediated signaling pathways. [71]. The observed downregulation of TREM-1 in our study suggests that maxadilan modulates inflammatory signaling to prevent uncontrolled immune activation, in line with other models where TREM-1 inhibition provided protective effects [72]. LPS-induced corneal inflammation initiates a cytokine cascade in which TNF-a and IL-6 play central roles in amplifying inflammatory responses and promoting tissue damage in the cornea [64,73–80]. The interplay between these cytokines sustains corneal inflammation [80]. By reducing both TNF-a and IL-6 by nearly 50%, maxadilan effectively disrupted this inflammatory loop, potentially contributing to reduced neutrophil recruitment, tissue damage, and apoptosis, thereby preserving corneal integrity [72,80,81]. Uncontrolled cytokine expression due to bacterial infection, can severely compromise corneal health by causing persistent inflammation, stromal degradation, and fibrosis [82,83]. Increased vascular permeability and immune cell infiltration further lead to tissue edema, delayed wound healing, and a weakened corneal barrier, which heightens the risk of secondary infections [84]. Over time, the persistent inflammatory response promotes pathological changes of the corneal surface, thereby increasing corneal opacity [62]. This transformation, as seen in inflammation caused by endogenous endophthalmitis, significantly impairs vision [85]. Based on Shioda et al. [86], it is well established that endogenous PACAP protects the cornea from keratinization in dry eye conditions. Recent findings suggest that activating the PAC1R may serve as a novel strategy to reduce inflammatory cytokines and protect against ocular inflammation-induced keratinization. In line with this, maxadilan completely abolished severe keratinization: while 50% of LPS + PBS-treated eyes developed Grade 3 keratitis, this was reduced to 0% with maxadilan (100% reduction). Furthermore, the proportion of mild keratitis (Grade 0–1) was detected in 33.2% in the LPS + PBS group and 83.3% in the LPS + maxadilan group, demonstrating a marked shift toward less severe epithelial pathology. Histological examination further confirmed the protective effects of maxadilan. In corneas treated with LPS without intervention, we observed stromal remodeling, epithelial thickening, and irregular cellular organization, consistent with previous observations described by Petrillo and colleagues as characteristics of early-stage keratinization [87]. These data correlate with those of Shioda and coworkers, who demonstrated the protective effects of endogenous PACAP against keratinization [86].

Beyond its role in inflammation and structural preservation, the broader applicability of PAC1R-based therapies is further supported by previous research on live bacterial infection models. Previous studies have demonstrated antimicrobial and immunomodulatory properties of PACAP, including activity against *Pseudomonas aeruginosa* and modulation of TLR/MyD88 signaling pathways [88,89]. These findings further support the broader therapeutic potential of PAC1R-mediated mechanisms. These findings underline PACAP’s dual function in antimicrobial defense and immunomodulatory—effects primarily mediated through PAC1R. Notably, TREM-1, which was significantly suppressed by maxadilan in our model, has also been identified as a key amplifier in *Pseudomonas aeruginosa*-induced keratitis, and its inhibition has been linked to improved corneal outcomes [72]. Based on this, it is possible that PAC1R activation could confer similar therapeutic benefits in ocular infections caused by live Gram-negative pathogens.

The systemic LPS model is suitable for investigating endogenous ocular inflammation, as systemic administration of lipopolysaccharide induces a strong inflammatory response resembling sepsis. This model reproduces key pathophysiological mechanisms such as cytokine release, blood–retinal barrier disruption, and immune-cell infiltration into ocular tissues. The simultaneous administration of maxadilan with LPS in this study primarily reflects a preventive intervention; therefore, future studies using delayed treatment protocols will be necessary to distinguish therapeutic efficacy from prophylactic effects. However, an important limitation is that the model does not involve live bacteria, and therefore it cannot fully reflect the complex host–pathogen interactions, bacterial replication, toxin production, and dynamic progression that characterize true infectious endophthalmitis. Despite this, the LPS model provides a highly reproducible and controllable system that allows detailed investigation of the inflammatory cascade and PAC1 receptor–mediated protective mechanisms.

A second limitation is the restricted temporal resolution of the study. Although routine histological assessment was performed 5 weeks after LPS administration, this single late endpoint does not allow for a detailed evaluation of the temporal dynamics of inflammatory progression and resolution. Intermediate phases—such as the shift from acute inflammation to tissue repair—were not monitored, preventing a full characterization of long-term outcomes, including potential fibrosis, neovascularization, epithelial remodeling, or chronic stromal alterations. Future studies incorporating longitudinal follow-up with multiple time points will be required to determine whether PAC1R activation exerts sustained protective effects beyond the acute phase.

Taken together, our findings and prior research highlight PAC1R activation as a promising therapeutic strategy for managing corneal inflammation associated with endophthalmitis and other Gram-negative bacterial ocular infections.

## Conclusion

In conclusion, our study provides compelling evidence that activation of PAC1R by the selective agonist maxadilan exerts protective effects on the cornea in a model of endotoxin-induced ocular inflammation. Administration of maxadilan significantly mitigated inflammation, reduced corneal edema, and preserved corneal integrity by modulating critical inflammatory cytokines and chemokines, thus effectively preventing pathological keratinization. Our results support further investigation of PAC1R activation as a therapeutic strategy for ocular inflammatory diseases, especially those driven by systemic or Gram-negative bacterial origins. These findings suggest that maxadilan may serve as a complementary therapeutic approach rather than a stand-alone treatment, while highlighting the PAC1 receptor as a potential target for future therapeutic development.

### Institutional review board statement

The study was conducted according to the guidelines of the Declaration of Helsinki and approved by the Institutional Review Board (or Ethics Committee) of the Animal Welfare Committee of the University of Pecs, and the National Scientific Ethical Committee on Animal Experimentation (ÁTET) at the Ministry of Agriculture, fully complying with the Decree No. 40/2013. (II. 14.) of the Hungarian Government and the EU Directive 2010/63/EU on the protection of animals used for scientific purposes (ethical permission numbers: BA02/2000–01/2022 (approval date: 2022.02.18), and ARVO Statement for the Use of Animals in Ophthalmic and Vision Research.

## Supporting information

S1 Fig Baseline anterior segment OCT B-scan images of the cornea before any treatment. This file presents representative anterior segment OCT B-scan images showing the baseline morphology of the central cornea before any LPS injection or intravitreal treatment. Panel A shows a Control + PBS eye with normal corneal structure and anterior chamber. Panel B displays a Control + Maxadilan eye, which similarly exhibits normal corneal morphology. Panels C and D show the baseline scans of the LPS + PBS and LPS + Maxadilan groups, respectively, both recorded prior to LPS administration. These images confirm that all corneas exhibited normal thickness and architecture before the experimental interventions. Abbreviations: PBS, phosphate-buffered saline; LPS, lipopolysaccharide; Maxa, maxadilan.
(TIF)
